# Plant Acyl-CoA-Binding Proteins—Their Lipid and Protein Interactors in Abiotic and Biotic Stresses

**DOI:** 10.3390/cells10051064

**Published:** 2021-04-30

**Authors:** Sze-Han Lai, Mee-Len Chye

**Affiliations:** School of Biological Sciences, The University of Hong Kong, Pokfulam, Hong Kong, China; szehan1@connect.hku.hk

**Keywords:** abiotic stress, acyl-CoA-binding proteins, biotic stress, lipids, protein interactors, stress signalling

## Abstract

Plants are constantly exposed to environmental stresses during their growth and development. Owing to their immobility, plants possess stress-sensing abilities and adaptive responses to cope with the abiotic and biotic stresses caused by extreme temperatures, drought, flooding, salinity, heavy metals and pathogens. Acyl-CoA-binding proteins (ACBPs), a family of conserved proteins among prokaryotes and eukaryotes, bind to a variety of acyl-CoA esters with different affinities and play a role in the transport and maintenance of subcellular acyl-CoA pools. In plants, studies have revealed ACBP functions in development and stress responses through their interactions with lipids and protein partners. This review summarises the roles of plant ACBPs and their lipid and protein interactors in abiotic and biotic stress responses.

## 1. Introduction

Lipids are crucial components in facilitating developmental and adaptation processes in all prokaryotes and eukaryotes. They form the primary structure of cell membranes, maintaining cellular fluidity and integrity, and provide energy during plant growth and development, including germination, organ differentiation and pollination [1,2,3]. Moreover, seed and seedling development require energy fuelled by lipids in the form of triacylglycerols (TAGs) [4,5,6]. Lipids also contribute to processes such as the establishment and maintenance of cellular polarity, and chlorophyll and carotenoid production in photosynthesis [7,8,9,10]. Previous studies have proven that plant lipids act as signal transducers in signalling pathways essential to development and responses in both biotic and abiotic stresses caused by pathogens, extreme temperatures, flooding, salinity, heavy metals, osmotic damage and wounding [11,12,13,14,15,16,17,18]. For instance, the rapid formation of aldehydes and alcohols from fatty acid (FA) hydroperoxides occurs upon wounding [19]. These derivatives from oxylipins can protect plants from fungal and insect attacks, and drive the expression of abiotic stress-associated genes [19]. Jasmonate (JA), a major phytohormone, can appear in several active forms including methyl JA (MeJA) and *cis*-jasmone, and these active metabolites act as signalling molecules, activating plant defensive genes when exposed to biotic and abiotic stresses [20]. Phosphatidic acid (PA), a minor class of glycerophospholipids, is a versatile lipid mediator that exhibits dynamic changes upon perceiving stress signals [21]. PA can modulate signalling and cellular processes through protein interactions that can cause several structural effects on target proteins such as membrane tethering, conformational change, competitive ligand binding and oligomerization [21]. Furthermore, epidermal lipids such as cutin, suberin and waxes, form physical barriers to control water loss and protect the plant against environmental threats [22,23,24,25,26,27,28,29].

There are eight categories of lipids, including fatty acyls, glycerolipids, glycerophospholipids, sphingolipids, saccharolipids, polyketides, sterol lipids and prenol lipids [30]. Glycerolipids, sphingolipids and sterols are the primary constituents of plant membranes [31]. In plants, de novo biosynthesis of lipids takes place in the plastids and endoplasmic reticulum (ER) [32]. Some of the acyl chains produced in the plastids [33] are used for galactolipid biosynthesis (the prokaryotic pathway), while the majority are transported to the ER for glycerolipid and extraplastidial membrane lipid biosynthesis (the eukaryotic pathway) [32,34]. In the ER, glycerol-3-phosphate is acylated with acyl-CoA exported from the plastids to generate PA [35,36]. PA can be converted to cytidyldiphosphate-diacylglycerol (CDP-DAG) by CDP-DAG synthase and subsequently to phosphatidylglycerol (PG) and phosphatidylinositol (PI) [37]. Alternatively, the dephosphorylation of PA to DAG can lead to the synthesis of phosphatidylcholine (PC), phosphatidylethanolamine (PE) and phosphatidylserine (PS) [37]. Phospholipids consist of a hydrophilic head containing a phosphate group and glycerol and two FA tails [38]. They form the main structural components of membranes, and are recognized as second messengers [39,40] in regulating plant growth and development, as well as cellular responses to environment or stress [41,42]. Many studies have shown that plants respond via phospholipid signalling to stress stimuli from salt, osmotic damage, temperature changes as well as pathogens [40,43,44,45,46,47].

Sphingolipids, a diverse class of lipids, constitute many structural combinations of sphingoid bases, the *N*-acylated fatty acid and the polar head group [48]. They can form complex sphingolipids in plants through the hydroxylation or desaturation of long-chain base (LCB) components and fatty acid chains [49]. The LCB and the ceramide can be present as phosphorylated or nonphosphorylated forms [50]. Plant sphingolipids are divided into four main classes namely ceramides, glycosylceramides (GlcCers), glycosylinositolphosphoceramides (GIPCs) and free LCBs, with GIPCs predominating in plant tissues [51]. Sphingolipids, well-studied in animal systems, have been implicated in many essential processes in plants including pollen development, signal transduction and responses to biotic and abiotic stresses [52].

Sterols are isoprenoids, and are another important integral membrane component having diverse functions in all eukaryotes [49]. Sterols regulate acyl chain ordering and, together with glycerolipids and sphingolipids, they reinforce the structure of cell membranes [53,54]. In addition, some sterols form special structures with sphingolipids known as microdomains or lipid rafts [55,56]. These structures regulate important processes such as signal transduction, protein trafficking and plant-pathogen interactions [54,57]. Plant sterols are mainly comprised of campesterol, stigmasterol, *β*-sitosterol and brassicasterol [49,58]. With exposure to abiotic stresses such as ultraviolet (UV) radiation, low temperature and drought, marked changes in sterol content are observed, indicating that sterols may regulate plant response to stresses by maintaining membrane stability [59,60,61,62]. In grapes, low-intensity UV induced the acclimation response to repair damaged membranes following sterol synthesis, whereas high-intensity UV triggered antioxidant production to counteract oxidative damage [60]. Cold stress, on the other hand, causes changes in lipid content and membrane fluidity, thus improving the mechanical adaption of plants to low temperature [61]. In dehydrated plants, an increase in steryl ester levels may be associated with membrane lipid metabolism to maintain membrane integrity [62].

Acyl-CoA-binding proteins (ACBPs) were first identified in rat brain as neuropeptides that prevent the binding of diazepam to the receptor of γ-aminobutyric acid (GABA) [63]. Diazepam is a benzodiazepine drug used in the treatment of anxiety disorders, muscle spasm, spasticity and alcohol detoxification [64]. It binds to the GABA receptor and causes the neuronal influx of chloride ions, thus decreasing the excitability of the neuron after hyperpolarisation of postsynaptic membranes [64]. Thus ACBP, also known as the diazepam-binding inhibitor, displaces diazepam from the GABA receptor binding site and suppresses the anxiolytic effects of diazepam or other benzodiazepines [65]. Later, ACBPs were discovered in other eukaryotes and some prokaryotes [66,67]. ACBPs bind to a variety of acyl-CoA esters with different affinities, implying that they play a role in the transport and maintenance of the acyl-CoA pool. ACBPs can recruit long-chain acyl-CoAs immobilised in multilamellar liposomes and form acyl-CoA/ACBP complexes [68]. Such complexes can then transport and donate acyl-CoAs to mitochondrial *β*-oxidation and microsomal glycerolipid synthesis [68]. When ACBPs are available in equimolar, or in excess, to long-chain acyl-CoAs, ACBP can sequester the synthesised acyl-CoA esters and prevent the inhibition of both acetyl-CoA carboxylase and the mitochondrial adenine nucleotide translocase caused by long-chain acyl-CoAs [69]. Hence, long-chain acyl-CoAs are protected from microsomal acyl-CoA hydrolases [69].

In yeast, reduction of ACBP levels coincides with differential gene expression of fatty acid and phospholipid biosynthesis, glycolysis and glycerol metabolism, and stress responses [70]. Roundworm (*Caenorhabditis elegans*) expresses seven ACBPs, and depletion of ACBP-1, ACBP-2 or ACBP-3 affects *β*-oxidation of fatty acids and intestinal lipid storage, indicating that ACBPs are important for lipid metabolism and storage in *C. elegans* [71]. The *C. elegans acbp-1* knockout mutant can mobilise lipid stores and increase fatty acid oxidation to a level similar to the wild type under starvation [71]. Knockdown of the *C. elegans* membrane-associated ACBP 1 (MAA-1) extended lifespan and improved resistance to heat and oxidative stresses, mediated by transcriptional regulator encoding a hypoxia inducible factor (HIF-1) [72]. Aging-induced proteotoxicity was also improved by the HIF-1 transcriptional activation of *small heat-shock proteins* (*sHSP*s), leading to longevity in *maa-1* mutants [72]. The stress responses caused by heat stress and oxidation in *C. elegans maa-1* mutants require further investigation. Moreover, an increasing number of studies on plant ACBPs have shown that ACBPs play different roles in development and stress responses by interacting with lipids and protein partners [73,74,75,76,77,78,79,80,81,82]. This review gathers current knowledge on the regulatory roles of plant ACBPs, together with lipids, in response to abiotic and biotic stresses.

## 2. Plant ACBPs

Plant ACBPs were first identified in *Brassica napus* L. (oilseed rape) as a 10-kDa homologue expressed in seeds, flowers and cotyledons [66]. It binds long-chain acyl-CoA esters [83], participates in acyl-CoA transport [84], maintains acyl-CoA pool [85], and regulates the activities of various enzymes including glycerol-3-phosphate acyltransferase [83], lysophosphatidylcholine acyltransferase [85] and lysophosphatidic acid acyltransferase [86]. The transport of acyl-CoA esters is important for the biosynthesis of lipids such as glycerolipids, ceramides and phospholipids, and studies have shown that the binding of phospholipids to ACBPs plays a role in plant growth and development as well as stress responses [76,78,87,88,89,90,91,92,93,94]. Following the discovery of BnACBP, similar 10-kDa ACBPs emerged in *Arabidopsis thaliana* [95], *Gossypium hirsutum* (cotton) [96], *Ricinus communis* (castor bean) [97], *Digitalis lanata* Ehrh. (Wolly Foxglove) [98], *Oryza sativa* (rice) [89], *Vernicia fordii* (tung tree) [99], *Vitis vinifera* (grape) [100], *Helianthus annuus* (sunflower) [101], *Elaeis guineensis* (oil palm) [102], *Zea mays* (maize) [103] and *Glycine max* (soybean) [104].

In plants such as Arabidopsis, rice, oilseed rape, oil palm, maize and soybean, ACBPs are classified into four main groups according to size and domains: Class I small ACBPs, Class II ACBPs containing ankyrin repeats, Class III large ACBPs and Class IV ACBPs containing kelch motifs (Table 1) [89,102,103,104,105]. Table 1 shows that Class I ACBPs range from 10 to 17 kDa, whereas the others comprised of a transmembrane domain, ankyrin repeats and/or kelch motifs, have molecular weights of 34 to 85 kDa [89,103]. Arabidopsis ACBPs are localised to the ER and plasma membrane (AtACBP1 and AtACBP2) [106,107], apoplast (AtACBP3) [108] and cytosol (AtACBP4 to AtACBP6) [87,109]. On the other hand, rice ACBPs are subcellularly localised to the cytosol (OsACBP1 to OsACBP3) [91], ER (OsACBP4) [91,110], apoplast (OsACBP5) [111] and peroxisomes (OsACBP6) [91]. In maize, transient expression of green fluorescent protein (GFP)-tagged Class I ZmACBP1 in *Nicotiana benthamiana* leaf epidermal cells revealed that ZmACBP1 was confined to the cytosol, Class II ZmACBP3 localised to the ER, whereas Class III and IV ZmACBP6 and ZmACBP7, respectively, were targeted to both the cytosol and the plasma membrane [103]. Oil palm Class II EgACBP2 contains an *N*-terminal transmembrane domain responsible for protein targeting to the plasma membrane, and two *C*-terminal ankyrin repeats which could mediate protein-protein interactions and other cellular activities [102]. Consistent with Protein Subcellular Localization Prediction Tool (PSORT) speculation, the sunflower Class I HaACBP6, which was transiently expressed in tobacco leaves, was localised to the cytosol and nucleus [101]. These results are summarised in Table 1.

Using isothermal titration calorimetry (ITC), it has been reported that all recombinant ACBPs (rACBPs) bind acyl-CoA esters with varying affinities; rAtACBP1 and rAtACBP3 displayed high affinity to very-long-chain (VLC) species [26,108,112], while rAtACBP3 to rAtACBP6 and rOsACBPs to medium-chain species [89,113]. All rAtACBPs and rOsACBPs bind long-chain acyl-CoA esters at different affinities [75,89,94,108,114,115]. Moreover, rACBPs were shown to bind phospholipids, all Arabidopsis rAtACBPs bind PC [78,87,112,115,116], and rAtACBP1, rAtACBP2 and rAtACBP3 bind PA, lysoPC, and PE, respectively [76,88,112,117]. In contrast, all rice rOsACBPs bind PA and PC [89]. Besides binding with high affinity to 16:0-CoA, 18:0-CoA and 18:1-CoA, sunflower Class I rHaACBP6 and Class II rHaACBP1 also bind to several PC species [101,118]. In addition to phospholipid binding, Arabidopsis ACBPs were shown to interact with protein interactors (Table 2). AtACBPs interact with various transcription factors that activate the gene expression for downstream abscisic acid (ABA) or ethylene responses upon perception of stress stimuli [73,77,80]. These transcription factors include ABA-RESPONSIVE ELEMENT BINDING PROTEIN1 (AREB1) [80], RELATED TO APETALA2.12 (RAP2.12) [77] and ETHYLENE-RESPONSIVE ELEMENT BINDING PROTEIN (AtEBP) [73]. Furthermore, AtACBPs bind enzymes for sterol or phospholipid metabolisms such as PHOSPHOLIPASE Dα1 (PLDα1) [78], STEROL C4-METHYL OXIDASE1-1 (SMO1-1) [119], SMO1-2 [81] and LYSOPHOSPHOLIPASE2 (LYSOPL2) [76,82], which are important for membrane stability and repair as well as plant development. Thus far, only AtACBP2 interacts with FARNESYLATED PROTEIN6 (AtFP6) which may be involved in phospholipid repair following heavy metal-induced lipid peroxidation [75]. Lipid binding of ACBPs and their protein-protein interactions are now known to be important in regulating abiotic and biotic stress responses [74,75,76,78,80,87,88,93,120,121,122,123,124,125,126,127,128], as well as plant development including embryogenesis [81,116,119], seed dormancy [78], seed germination and development [78,80,129,130,131], cuticle development [25,26], pollen growth [132] and senescence [112,117].

## 3. Membrane Lipids and ACBPs in Abiotic Stress Signalling

**Figure 1:**
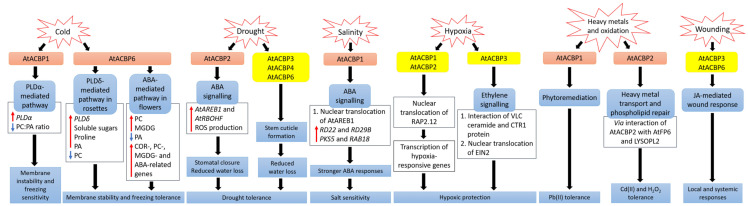
Signalling pathways associated with acyl-CoA-binding proteins (ACBPs) following abiotic stresses in *Arabidopsis thaliana*. In transgenic Arabidopsis Class II AtACBP1-overexpressors (OEs), *PLDα1* was induced upon cold stress, causing a decrease in the ratio of PC to PA leading to membrane instability and freezing sensitivity [88]. In contrast, transgenic Arabidopsis Class I AtACBP6-OEs were conferred freezing tolerance via the PLDδ-mediated pathway in rosettes and the ABA-mediated pathway in flowers, resulting in changes in lipids, sugars and stress-related genes [87,123]. During drought, transgenic Arabidopsis Class II AtACBP2-OEs exhibited elevated *AtAREB1* and *AtRBOHF* expression which led to ROS production, subsequent stomatal closure and reduced water loss [90]. Proper stem cuticle development conferred by Class I AtACBP6, Class III AtACBP3 or Class IV AtACBP4 protects wild-type Arabidopsis from water loss [25]. Under high salinity, *AtACBP1* and *AtAREB1* expression were upregulated in wild-type seeds [80]. The overexpression of AtACBP1 in transgenic Arabidopsis triggers nuclear translocation of AtAREB1, leading to the induction of stress marker genes (*RD22* and *RD29B*) and AtAREB1 target genes (*PKS5* and *RAB18*), thereby promoting stronger ABA responses during seed germination and seedling establishment [80]. When wild-type Arabidopsis undergoes hypoxia, the RAP2.12 transcription factor bound to AtACBP1 or AtACBP2, translocates to the nucleus and activates hypoxia-responsive gene transcription, conferring hypoxic protection [77,128,134,139]. Another hypoxic tolerance pathway involves the interaction of unsaturated VLC ceramide and the CTR1 protein with subsequent nuclear translocation of EIN2, resulting in the activation of CTR1-mediated ethylene signalling [137]. AtACBP1 is involved in phytoremediation and its overexpression in transgenic Arabidopsis confers Pb(II) tolerance [135]. AtACBP2 can interact with AtFP6 or LYSOPL2, mediating heavy metal transport and phospholipid repair respectively, and hence transgenic Arabidopsis AtACBP2-OEs were resistant to Cd(II) and Cd(II)-induced oxidative stress [75,76,82]. On wounding, the up-regulation of *AtACBP3* and *AtACBP6* expression in the wild type suggested their involvement in JA-mediated local and systemic wound responses [125,126]. Orange and yellow boxes indicate transgenic Arabidopsis AtACBP-OEs and wild-type Arabidopsis AtACBPs respectively, used in studies on abiotic stress. Blue boxes represent the signalling pathways. White boxes indicate the molecular events that occur along the signalling pathway. Red and blue arrows indicate increase and a decrease, respectively. Black arrows denote the flow of events. ABA, abscisic acid; ACBP, acyl-CoA-binding protein; AREB1, ABA-RESPONSIVE ELEMENT BINDING PROTEIN1; FP6, FARNESYLATED PROTEIN6; COR, COLD-RESPONSIVE; CTR1, CONSTITUTIVE TRIPLE RESPONSE1; EIN2, ETHYLENE-INSENSITIVE2; JA, jasmonic acid; LYSOPL2, LYSOPHOSPHOLIPASE2; MGDG, monogalactosyldiacylglycerol; PA, phosphatidic acid; PC, phosphatidylcholine; PKS5, PROTEIN KINASE SOS2-LIKE5; PLD, PHOSPHOLIPASE D; RAB18, RESPONSIVE TO ABA18; RAP2.12, RELATED TO APETALA2.12; RBOHF, RESPIRATORY BURST OXIDASE HOMOLOG F; RD, RESPONSIVE TO DESSICATION; ROS, reactive oxygen species; VLC, very-long-chain.

Plants are sessile, and therefore possess signalling and adaptive mechanisms to counteract abiotic and biotic stresses including cold, drought, salinity, oxidation, heavy metals, hypoxia and pathogen attack. Given the importance of plant ACBPs in development and stress responses, the roles of all Arabidopsis and rice ACBPs at different stages of plant growth were previously summarized by Du et al. [138] and are now updated (Table 2). Studies on the binding by ACBPs of acyl-CoA esters, membrane lipids and protein interactors have provided insights into the mechanistic events that occur when plants are exposed to various abiotic stresses (Figure 1).

### 3.1. Cold Stress

AtACBP6-overexpressing (AtACBP6-OE) transgenic Arabidopsis rosettes and flowers are freezing tolerant (Figure 1) [87,123]. Northern-blot and Western-blot analyses showed that the expression of *AtACBP6* and its protein in the wild type was induced at 48 h after 4 °C cold treatment [87]. The *atacbp6* mutant showed increased sensitivity to freezing temperature (−8 °C) in contrast to the AtACBP6-OE plants [87]. Lipid profiles of rosettes upon freezing treatment of AtACBP6-OE transgenic Arabidopsis recorded decreases in PC and increases in PA, over the wild-type plants [87]. Furthermore, in vitro filter-binding assays revealed that rAtACBP6 binds PC, but not PA or lysoPC, suggesting a role for AtACBP6 in phospholipid metabolism in Arabidopsis [87]. On the other hand, in transgenic Arabidopsis AtACBP6-OE flowers, PC and monogalactosyldiacylglycerol (MGDG) levels were elevated while PA decreased [123]. In AtACBP6-OE rosettes, *PHOSPHOLIPASE Dδ* (*PLDδ*) was upregulated in the absence of *COLD-RESPONSIVE* (*COR*)-related gene induction [87], while flowers showed increased expression of *COR*-related genes and their transcription factors (*C-repeat binding factors* (*CBFs*), *INDUCER OF CBF EXPRESSION1* (*ICE1*) and *MYB15*), PC-related genes, MGDG-related genes and ABA-related genes [123]. These results suggest a differential mechanism of freezing tolerance conferred by AtACBP6 in rosettes and flowers, possibly mediated by soluble sugar and proline accumulation and the ABA signalling pathway, respectively (Figure 1) [123].

Besides Class I AtACBP6, Arabidopsis Class II AtACBP1 also plays a role in freezing tolerance (Figure 1) [88]. AtACBP1-OE transgenic Arabidopsis plants were more cold-sensitive, accompanied by PC reduction and PA elevation, while *atacbp1* plants were better protected from freezing arising from an increase in PC and a reduction in PA [88]. Although AtACBP1 and AtACBP6 belong to the same protein family, they play distinctive roles in cold tolerance. In vitro binding of rAtACBP1 to PA indicated possible enhanced PA interaction in AtACBP1-OE plants [78,88]. PLDα1, an important enzyme that catalyses the conversion of PC to PA, showed a higher gene expression in AtACBP1-OE plants than in *atacbp1* [88]. In contrast, *PLDδ* expression decreased in the AtACBP1-OEs but increased in *atacbp1* [88]. As AtACBP1 is localised to the ER and plasma membrane, it may maintain a membrane-associated PA pool through PA binding, thereby regulating the expression of PLDα1 and PLDδ [88].

Other than AtACBPs, grape *VvACBP* was upregulated in leaves upon cold and heat shock stresses in comparison to the nontreated control [100]. In maize, the expression levels of *ZmACBP2*, *ZmACBP3*, *ZmACBP5* and *ZmACBP6* were induced by cold stress while *ZmACBP1*, *ZmACBP4*, *ZmACBP7*, *ZmACBP8* and *ZmACBP9* mRNA levels declined after cold treatment [103]. These changes in expression levels depicted the potential roles of ZmACBPs in cold stress response which remain to be further elucidated. RNA-seq data analysis of the expression of soybean *GmACBPs* showed that only Class IV *GmACBP11* was downregulated at 24 h after cold stress, whereas other *GmACBPs* displayed a lack of significant changes of expression in comparison to the nontreated control [104].

### 3.2. Drought Stress

Drought stress has received massive attention as it threatens worldwide crop production. ABA is a plant hormone that plays vital roles in many physiological processes including responses to abiotic stresses such as drought and salinity [140]. Under water deficiency, plants produce adaptive responses through the expression of various genes upon elevation of ABA [141,142]. Many signal transducers have been reported to participate in ABA signalling, including PA, diacylglycerol (DAG), phosphoinositides, reactive oxygen species (ROS), cyclic adenosine 5′-diphosphate ribose, sphingosine 1-phosphate and calcium [143,144,145,146,147,148,149,150,151,152].

Class II membrane-associated AtACBP2 responds to drought stress via ABA signalling (Figure 1) [90]. *AtACBP2* expression was induced by ABA and drought treatment in wild-type Arabidopsis seedlings [90]. On top of that, transgenic Arabidopsis AtACBP2-OEs showed better drought tolerance than the wild type, whereas the *atacbp2* mutant plants were more sensitive after drought treatment [90]. ABA-signalling genes including *AREB1* and *RESPIRATORY BURST OXIDASE HOMOLOG F* (*AtRBOHF*) were upregulated in AtACBP2-OE before and after ABA treatment while *AtRBOHD* and *ABA DEFICIENT2* (*ABA2*) increased only after ABA treatment. These results support the role of AtACBP2 in ABA signalling and hence in drought tolerance, as characterized by stomatal closure and reduced water loss [90].

It has been suggested that AtACBP3, AtACBP4 and AtACBP6 can regulate drought tolerance through stem cuticle formation (Figure 1) [25]. Transmission electron microscopy (TEM) showed that the leaves of the *atacbp3*, *atacbp4* and *atacbp6* mutants each had an abnormal and more permeable cuticle in comparison to the wild type, resulting in water loss after drought stress [25]. Furthermore, marked changes of cuticular wax and cutin monomer profiles in *atacbp3*, *atacbp4* and *atacbp6* single mutant plants depicted that AtACBPs play an important role in cuticle formation as well as in drought tolerance [25]. In soybean, expression profiles of roots were analysed by RNA-seq following dehydration stress [153]. Data mining of *GmACBP* expression by Azlan et al. [104] revealed that Class II (*GmACBP3* and *GmACBP4*), Class III (*GmACBP7*) as well as Class IV (*GmACBP9*) were induced, suggesting that these GmACBPs play a role in drought response.

### 3.3. Salinity Stress

High salt in soil is detrimental to plant growth and development, and this in turn severely affects the crop yield worldwide. Salt stress can induce other stresses including osmotic stress, ionic stress and oxidative stress [154,155]. Osmotic stress arises from the reduction of water potential due to high amount of salt at the root surface, leading to a reduction in water uptake by the plant [156]. Ionic stress occurs as there is excessive uptake of sodium (Na^+^) and chloride (Cl^+^) ions by plant roots, which eventually accumulate in leaves [157]. Besides, ROS production also increases upon exposure to salt stress, causing oxidative stress in plants [158,159,160,161,162,163].

Salt sensing and signalling are complex. One of the early salt-signalling components are phospholipids, including polyphosphoinositides and PA [164,165]. PI signalling triggers the biosynthesis of phosphoinositides and JA-related proteins upon salt stress and can rapidly remodel soybean lipid composition for stress adaption [166]. Under salt stress, Na^+^ homeostasis is regulated by the SALT OVERLY SENSITIVE (SOS) pathway whereby Na^+^ influx promotes PLDα1 enzyme activity, causing a rise in PA levels [167]. Acting as a signal relay, PA activates MITOGEN-ACTIVATED PROTEIN KINASE6 (MPK6) which then phosphorylates SOS1, a potential intracellular Na^+^ sensor [168,169,170]. Several *pld* mutants exhibit enhanced sensitivity to salt stress [171].

*ChACBP1*, isolated from the algae (*Chlorella* sp.) JB6, was induced under various abiotic stresses including salinity, oxidation, heavy metals and cold stresses [92]. Given the binding of rChACBP1 protein to PC and the improved tolerance of yeast and Arabidopsis overexpressing *ChACBP1* to abiotic stresses, these responses may be mediated through phospholipid metabolism [92]. Following NaCl or mannitol treatment of Arabidopsis seeds, the expression of *AtACBP1* and its protein partner *AtAREB1* were upregulated over the water-treated control [80]. The overexpression of AtACBP1 rendered higher sensitivity of transgenic Arabidopsis to NaCl or mannitol treatment during seed germination and seedling establishment over the wild type, whereas the *atacbp1* mutant was less sensitive during seed germination but not seedling establishment (Figure 1) [80]. In transgenic Arabidopsis DsRed-AtAREB1/AtACBP1-OEs, the overexpression of AtACBP1 led to nuclear translocation of DsRed-AtAREB1 [80]. Salt and osmotic stress marker genes (*RD22* and *RD29B*) and AtAREB1 target genes (*PKS5* and *RAB18*) were also induced in AtACBP1-OEs [80]. These results suggested that enhanced AtAREB1 production in AtACBP1-OEs promotes stronger ABA responses during seed to seedling transition when AtAREB1 is released from AtACBP1 to enter the nucleus (Figure 1) [80]. A recent study revealed that the overexpression of OsACBP4 and AtACBP2 conferred salt resistance in both transgenic rice and Arabidopsis [94]. Four salinity-responsive elements in the *OsACBP4* 5′-flanking region were confirmed to interact with nuclear proteins from salt-treated rice [94]. On top of that, the up-regulation of genes encoding acyl-CoA synthase under salt stress and the binding of rOsACBP4 to long-chain acyl-CoA esters suggested that OsACBP4 may regulate salinity responses via lipid metabolism [94].

A recent study by Zhu et al. [103] showed that Class I *ZmACBP1* and Class II *ZmACBP3* gene expression was induced after NaCl or mannitol treatment. Transgenic Arabidopsis overexpressing ZmACBP1 and ZmACBP3 exhibited better growth and longer roots in comparison to the vector control [103]. The expression levels of the lipid metabolic genes (*FAD2*, *DGAT*, *PLA2*, *PLC3*, and *ACX*) and stress-responsive genes (*COR47*, *AREB1*, *RAB*, *ABI1*, *RD29A*, and *RD29B*) under NaCl or mannitol significantly increased in ZmACBP3-OEs compared to the wild type [103]. These results suggested that *ZmACBP3* overexpression may enhance stress tolerance through changes in lipid metabolism which led to the induction of stress-responsive genes [103]. In soybean response to NaCl stress, in silico analysis of *GmACBP* expression from RNA-seq data exhibited induction of Class II *GmACBP3*, Class III *GmACBP7* and Class IV *GmACBP9*, but decreases in Class I *GmACBP2* and Class IV *GmACBP10* [104]. As only Arabidopsis and rice Class II ACBPs have been reported in the NaCl response, the greater increase of Class III *GmACBP7* than Class II *GmACBP3* expression implied different roles for GmACBPs in soybean [104].

### 3.4. Hypoxic Stress

Plants need oxygen for respiration. Hypoxia happens when plants encounter oxygen deprivation, usually arising from flooding and soil waterlogging. Plants regulate their oxygen-sensing ability by transcription factors belonging to group VII of the ETHYLENE-RESPONSE FACTORS (ERF-VIIs) which are protected against proteasomal degradation only under hypoxia [77]. The stabilized ERF-VIIs can translocate to the nucleus and bind the HYPOXIA-RESPONSIVE PROMOTOR ELEMENT (HRPE) to drive the transcription of anaerobic genes [172]. ERF-VII transcription factor, AtEBP interacts with AtACBP2 via the ankyrin repeats although AtEBP is colocalised to the nucleus, whereas AtACBP2 is found on the plasma membrane [73]. Under aerobic conditions, RAP2.12 interacts with AtACBP1 and AtACBP2 at the plasma membrane, preventing its translocation to the nucleus and protecting it from *N*-end rule degradation [77]. When hypoxia arises, RAP2.12 is transported to the nucleus to activate the transcription of hypoxia-responsive genes (Figure 1) [139]. Polyunsaturated 18:3-CoA was proven to regulate the release of RAP2.12 from the plasma membrane upon hypoxia [128]. Upon submergence, wild-type Arabidopsis significantly accumulated polyunsaturated 18:3-CoA [128]. Confocal microscopy and immunoblot analysis showed that 18:3-CoA promoted stronger stabilization of RAP2.12-GFP, HYPOXIA RESPONSIVE ERF 1 (HRE1)-GFP and RAP2.3-GFP fusions [128]. In vitro pull-down assays revealed that both 18:0- and 18:3-CoAs suppress the interaction of AtACBP1 and ERF-VII, suggesting that 18:3-CoA can modulate the dissociation of the AtACBP1-ERF-VII complex when hypoxia arises [128]. Moreover, 18:3-CoA treatment of *atacbp1* AtACBP2-RNAi lines indicates that AtACBP1 and AtACBP2 are important for the 18:3-CoA-induced stabilization of RAP2.12 and induction of hypoxia-responsive genes [128]. In addition, cellular energy depletion following hypoxia increased 18:1-CoA levels, triggering the dissociation of AtACBP1-bound RAP2.12 and its subsequent nuclear translocation for the activation of hypoxic gene transcription [134].

Other than Class II AtACBPs, AtACBP3 also plays a role in hypoxic response in Arabidopsis through binding of VLC acyl-CoA esters and regulation of fatty acid metabolism such as unsaturated VLC ceramides [136]. The interaction of unsaturated VLC ceramide with the CONSTITUTIVE TRIPLE RESPONSE1 (CTR1) protein promoted nuclear translocation of ETHYLENE-INSENSITIVE2 (EIN2), triggering CTR1-mediated ethylene signalling for hypoxic protection in Arabidopsis (Figure 1) [137]. Besides ceramides and acyl-CoAs, other lipids including phospholipids, galactolipids, oxylipins, wax and cutin are important in plant hypoxic responses [173]. Upon submergence, total PC, PE and phosphatidylglycerol (PG) content declined but phosphatidylserine (PS), PA, PI, oxidized lipid, ceramide and hydroxyceramide levels increased significantly [136,137]. Moreover, significant increase of oxidized galactolipids [MGDG and digalactosyldiacylglycerol (DGDG)] and phospholipids (PC, PE and PG), arabidopsides and malondialdehyde (MDA), implied that an oxidative burst occurs during hypoxia or posthypoxic reoxygenation, leading to significant lipid peroxidation [128,137,174]. In addition, transcriptomic analyses have shown changes in the expression of genes encoding proteins essential for the ceramide and sphingolipid LCB biosynthesis [137], lipid transfer, and wax and cutin transport during submergence [27]. Moreover, JA biosynthesis genes were enhanced upon postsubmergence reoxygenation, implicating that oxylipins may modulate the posthypoxic reoxygenation response in plants [174].

### 3.5. Heavy Metal and Oxidative Stresses

Heavy metals such as lead [Pb(II)], cadmium [Cd(II)] and zinc [Zn(II)] are major pollutants threatening the environment and living organisms. Therefore, several studies have been performed to investigate the role of AtACBPs in response to heavy metal stresses [75,135]. Using metal-chelate affinity chromatography and fluorescence analysis using dansyl aziridine-labelled proteins, rAtACBP1 was reported to bind Pb(II) [135]. The overexpression of *AtACBP1* in transgenic Arabidopsis showed better tolerance to Pb(II) stress, whereas the *atacbp1* mutant was more sensitive to Pb(II) (Figure 1). Accumulation of Pb(II) in the shoots of *AtACBP1*-overexpressing plants suggested a possible role of AtACBP1 in Pb(II) phytoremediation [135]. Besides *AtACBP1*, the expression of *AtACBP4* was also induced by Pb(II) in both Arabidopsis shoots and roots [124]. When transgenic *Brassica juncea* expressing *AtACBP1* and *AtACBP4* were grown in Pb(II)-containing media, Pb(II) accumulated in the cytosol of root tips and the vascular tissues, further corroborating to the function of AtACBPs in phytoremediation [124].

AtACBP2, on the other hand, is responsive to Cd(II). Although there was no accumulation of heavy metals in *AtACBP2*-overexpressing plants, the overexpression of *AtACBP2* enhanced tolerance to Cd(II) and oxidative stress (hydrogen peroxide, H_2_O_2_) in transgenic Arabidopsis (Figure 1) [75]. In the plasma membrane, AtACBP2 interacts via its ankyrin repeats with AtFP6, which has a metal-binding motif [75]. In Arabidopsis roots, *AtFP6* expression was induced after Cd(II) treatment [75]. The overexpression of AtFP6 conferred better Cd(II) resistance than the wild type, possibly by mediating heavy metal transport in plants [75]. LYSOPL2, another protein interactor of AtACBP2, is an intermediate of phospholipid metabolism and detoxifies lysoPC [76]. *LYSOPL2* expression was induced by Zn(II) and H_2_O_2_ in Arabidopsis. The overexpression of *LYSOPL2* in Arabidopsis exhibited enhanced tolerance to Cd(II) and H_2_O_2_ in comparison to the wild type, suggesting the involvement of LYSOPL2 in phospholipid repair following metal-induced lipid peroxidation (Figure 1) [76]. Possibly, the efficiency of membrane repair could be improved by the formation of an AtLYSOPL2-AtACBP2 complex, facilitated by lysoPC binding to AtACBP2 [82].

### 3.6. Wounding

In plants, wounding results following biotic attack (herbivores, insects and pathogens), mechanical damage or weather-induced damage, which may culminate in the entry of pathogens and nutrient loss. Mechanical injury triggers the transduction of mobile signals in the plants, leading to localised responses at the wound sites (local response) and distal responses in the undamaged tissues (systemic response) [175]. Cell wall-derived oligogalacturonides (OGs) and a polypeptide systemin are well-characterized wounding signals [176]. Upon wounding, systemin interacts with a cell-surface receptor to trigger several signalling events, including the release of linolenic acid from plant cell membranes and its conversion to 12 oxo-phytodienoic acid (OPDA) and JA [175,177,178]. The accumulation of JA in wounded plants subsequently activates various defence genes encoding proteinase inhibitor, thionin and enzymes involved in secondary metabolism [179].

Both Class I AtACBP6 and Class III AtACBP3 are involved in the local and systemic wound responses in Arabidopsis [125,126]. AtACBP6 and AtACBP3 proteins are localised to the companion cells, sieve elements and phloem [125,126]. On wounding, *AtACBP6* and *AtACBP3* were induced in Arabidopsis [125,126]. In comparison to *atacbp3* and *AtACBP3*-RNAi plants, wound-responsive JA marker genes such as *JASMONATE ZIM-DOMAIN10, VEGETATIVE STORAGE PROTEIN2* and *LIPOXYGENASE2*, were upregulated more significantly in locally wounded and systemic wild-type leaves [126]. Besides, lower levels of MeJA and oxylipin-related FAs, including C18:2-FA and C18:3-FA, were observed in *atacbp3* and *AtACBP3*-RNAi over wild-type phloem exudates [126]. ITC data showed that rAtACBP3 binds medium and long-chain acyl-CoA esters but not MeJA, suggesting that AtACBP3 maintains FA pool but does not transport MeJA in the phloem [126]. Taken together, the evidence indicated that AtACBP3, a phloem-mobile protein, possibly regulates JA-mediated local and systemic wound responses by its binding to acyl-CoA esters (Figure 1). Besides *AtACBP3* and *AtACBP6*, rice *OsACBP5* and *OsACBP6,* as well as several maize *ZmACBPs* (*ZmACBP1*, *ZmACBP2*, *ZmACBP5* and *ZmACBP6*), were rapidly induced after wound treatment [89,103]. However, their specific roles in wound response remain to be elucidated.

## 4. Membrane Lipids and ACBPs in Pathogen Defense

**Figure 2:**
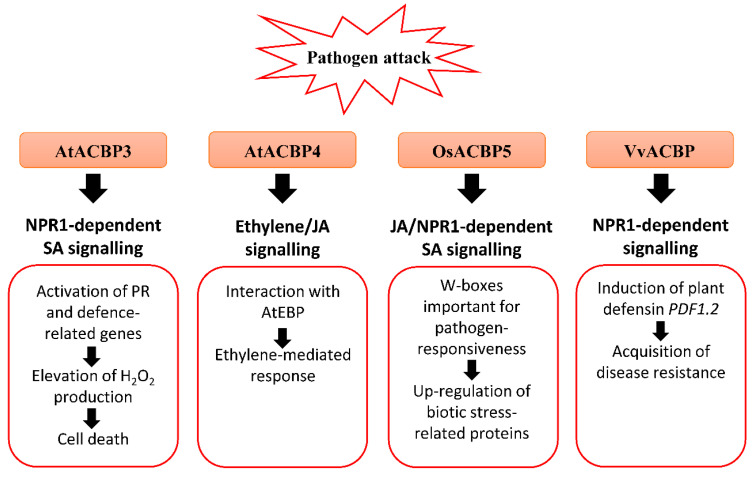
Biotic stress-related signalling pathways associated with acyl-CoA-binding proteins (ACBPs) in plants. Upon pathogen infection, the overexpression of Class III AtACBP3 in transgenic *Arabidopsis thaliana* led to constitutive activation of pathogenesis-related (PR) genes including *PR1*, *PR2* and *PR5*, elevated H_2_O_2_ production and eventually cell death [121]. AtACBP3 plays a distinct role in the plant defence response against necrotrophic and biotrophic pathogens as transgenic Arabidopsis AtACBP3-overexpressors (OEs) were protected against the biotrophic pathogen (*Pseudomonas syringae* pv *tomato* DC3000) but not the necrotrophic pathogen (*Botrytis cinerea*) [121]. In wild-type Arabidopsis, the expression of Class IV *AtACBP4* and *AtEBP* encoding a protein interactor of AtACBP4 were reported to be induced by *B. cinerea* infection, and ethylene precursor 1-aminocyclopropane-1-carboxylic acid (ACC) and methyl jasmonate (MeJA) treatments, suggesting that *AtACBP4* and *AtEBP* are mediated by ethylene and/or JA signalling [74]. Rice Class III OsACBP5 protects transgenic Arabidopsis and rice plants against hemibiotrophs and biotrophs via NPR1-dependent SA signalling, and necrotrophs by JA signalling [93,127]. The *OsACBP5* 5′-flanking region contains W-boxes which were verified in pathogen-responsiveness of *OsACBP5* [93]. Proteomic studies showed that eleven biotic stress-related proteins were upregulated by *Rhizoctonia solani* infection in transgenic Arabidopsis OsACBP5-OEs [127]. Grape Class III VvACBP conferred resistance to *P. syringae* and *Colletotrichum higginsianum* in transgenic Arabidopsis, possibly through the NPR1-mediated pathway following induction of *PDF1.2*, the gene encoding plant defensin [100]. Orange boxes represent the named ACBPs involved in the pathogen response. White boxes indicate the molecular events that occur along the signalling pathway. Black arrows denote the flow of signalling events. ACBP, acyl-CoA-binding protein; EBP, ETHYLENE-RESPONSIVE BINDING PROTEIN; JA, jasmonic acid; NPR1, NONEXPRESSOR OF PR-1; PR, pathogenesis-related; SA, salicylic acid.

Under the natural environment, plants are always exposed to a variety of bacterial and fungal pathogens. Several AtACBPs, such as AtACBP1, AtACBP3, AtACBP4 and AtACBP6, and Class III OsACBP5, have been reported to participate in plant defence against infections caused by bacterial and fungal pathogens (Figure 2) [25,26,93,121,127]. *AtACBP3* expression was induced in wild-type Arabidopsis following pathogen infection (*Pseudomonas syringae* pv *tomato* DC3000 and *Botrytis cinerea*) and treatments using pathogen elicitors (arachidonic acid) and defence-related phytohormones [1-aminocyclopropane-1-carboxylic acid (ACC), MeJA and salicylic acid (SA)] [121]. An S-box (TTTAA) regulatory element identified at the *AtACBP3* 5′-flanking region was verified by electrophoretic mobility shift assay (EMSA) to bind nuclear proteins from pathogen-infected Arabidopsis leaves [122]. In addition, overexpression of *AtACBP3* led to constitutive activation of pathogenesis-related (PR) genes, including *PR1*, *PR2* and *PR5*, H_2_O_2_ production and cell death (Figure 2) [121]. Following *P. syringae* pv *tomato* DC3000 infection, a lower bacterial count signified better protection of AtACBP3-OEs against the pathogen in comparison to the wild type and *atacbp3* mutant [121]. To determine whether the upregulation of *PR* genes is associated with the NONEXPRESSOR OF PR-1 (NPR1) or CORONATINE-INSENSITIVE1 (COI1) signalling pathway, transgenic Arabidopsis of the AtACBP3-OE*npr1* line was subject to *P. syringae* treatment [121]. Results showed that the *PR* genes were downregulated in AtACBP3-OE*npr1* and they no longer exhibit enhanced resistance to *P. syringae* infection, implying that the pathogen protection of AtACBP3-OEs is mediated by the NPR1 signalling pathway [121]. As AtACBP3-OEs were more susceptible to necrotrophic fungus *B. cinerea* infection compared to *atacbp3*, AtACBP3 is believed to play a differential role in the plant defence response against necrotrophic and biotrophic pathogens [121]. Apart from abiotic stress, grape VvACBP which belongs to the same Class III as AtACBP3, also plays a role in pathogen defence (Figure 2) [100]. The expression of VvACBP in transgenic Arabidopsis conferred resistance to *P. syringae* pv *tomato* DC3000 and *Colletotrichum higginsianum* upon infection [100].

The expression of Class IV *AtACBP4* and its protein interactor *AtEBP* were elevated following *B. cinerea* infection, as well as the ethylene precursor ACC and MeJA treatments (Figure 2) [74]. The interaction of AtACBP4 and AtEBP, as confirmed by yeast two-hybrid and coimmunoprecipitation, suggests that plant pathogen defence may be mediated by ethylene and/or JA signalling [74]. Another study revealed that *atacbp3*, *atacbp4* and *atacbp6* single mutants exhibited a defective cuticle that resulted in compromised systemic acquired resistance (SAR) to fungal (*B. cinerea* and *C. higginsianum*) and bacterial (*P. syringae*) pathogens [25]. Furthermore, AtACBP1 which is also important for stem cuticle formation, is suggested to confer resistance to *B. cinerea* [26].

Apart from AtACBPs, recent studies have depicted that Class III OsACBP5, the homologue of AtACBP3, protects rice plants against representative necrotrophic (*Rhizoctonia solani* and *Cercospora oryzae*), hemibiotrophic (*Magnaporthe oryzae* and *Fusarium graminearum*) and biotrophic (*Xanthomonas oryzae*) phytopathogens (Figure 2) [93]. Transgenic rice OsACBP5-OEs demonstrated stronger disease resistance against all pathogens tested [93]. In addition, enhanced resistance of OsACBP5-OEs against hemibiotrophs and biotrophs is mediated by SA signalling, while that against the necrotrophic pathogen *R. solani* is regulated by JA signalling [93]. In the *OsACBP5* 5′-flanking region of the four W-boxes (pathogen-responsive *cis*-elements) identified, EMSAs showed that two of them bound nuclear proteins from wild-type rice infected with *R. solani*, *C. oryzae*, *M. oryzae* and *X. oryzae* [93]. Furthermore, transgenic rice expressing the construct of the *OsACBP5* 5′-flanking region containing both these W-boxes fused to the gene encoding β-glucuronidase (GUS) exhibited higher GUS activity upon SA, MeJA or *R. solani* treatment compared to the promoter deletion lacking both W-boxes [93]. These results suggest that the W-boxes are important in the pathogen-responsiveness of *OsACBP5*.

Both Lipidex assays and ITC showed that rOsACBP5 binds to 18:3-CoA esters, suggesting that 18:3-FA, a precursor for JA biosynthesis, plays a role in basal defence against fungal pathogens [89,93]. Furthermore, proteomic analysis revealed that eleven biotic stress-related proteins were upregulated by *R. solani* infection in transgenic Arabidopsis OsACBP5-OEs. These proteins include cell wall-related proteins such as FASCILIN-LIKE ARABINOGALACTAN-PROTEIN10 (FLA10), LEUCINE-RICH REPEAT EXTENSIN-LIKE PROTEINS (LRX4 and LRX5), XYLOGLUCAN ENDOTRANSGLUCOSYLASE/HYDROLASE PROTEIN4 (XTH4) and PECTINESTERASE INHIBITOR18 (PME18), proteins involved in glucosinolate (GSL) degradation including GDSL-LIKE LIPASE23 (GLL23), EPITHIOSPECIFIER MODIFIER1 (ESM1), MYROSINASE1, MYROSINASE2, NITRILASE1 (NIT1), and a protein involved in JA synthesis, ALLENE OXIDE CYCLASE2 (AOC2), suggesting their potential in protection against *R. solani* [127].

Upon *Phakopsora pachyrhizi* fungal infection of soybean, seven *GmACBP*s comprising all four classes were detected in microarray data analysis [104]. The expression of Class I *GmACBP2*, Class II *GmACBP4*, Class III *GmACBP5* and *GmACBP6*, and Class IV *GmACBP11* declined after 6 h post inoculation (hpi) of soybean with avirulent Hawaii 94-1 and virulent Taiwan 80-2 strains [104]. In addition, *Phytophthora sojae* infection caused an induction of only Class IV *GmACBP9* at 72 hpi, but a reduction of other *GmACBPs* such as Class I *GmACBP2*, Class III *GmACBP5*, *GmACBP6* and *GmACBP7* as well as Class IV *GmACBP11* after 48 dpi [104]. Such differential changes in *GmACBPs* compared to other Class III *ACBPs* such as *AtACBP3*, *VvACBP* and *OsACBP5* in pathogen response, suggested differential roles of GmACBPs in plant-pathogen interactions.

## 5. Conclusions and Perspectives

Studies on ACBPs in the past two decades have strongly implicated their roles in the regulatory mechanisms of development and stress responses. All classes of ACBPs are known to play a role in abiotic and biotic stress signalling, although the detailed mechanistic events remain to be further elucidated. Through phospholipid and acyl-CoA ester binding, AtACBPs and OsACBPs are involved in stresses arising from drought [22,81], adverse temperatures [87,88,123], salinity [80], oxidation [76], hypoxia [73,77,128,136,137], heavy metals [75,76,120,124], wounding [125,126] and pathogens [74,93,100,121,122,127]. Moreover, the identification of ACBP protein interactors including AREB1 [80], RAP2.12 [77,128,134], AtEBP [73,74], AtFP6 [75], and LYSOPL2 [76,82], also lead to a better understanding of the signalling pathways of stress responses.

Taken together, dicot AtACBPs have been widely studied in relation to their acyl-CoA and phospholipid binding properties, protein-protein interaction and the resultant signalling cascades of abiotic and biotic stress responses. However, the roles of the monocot OsACBPs and ZmACBPs, as well as the emerging leguminous GmACBPs, remain to be further investigated given that preliminary studies have shown stress-induced changes in ACBP expression [89,103,104]. Thus far, only Class II OsACBP4 and Class III OsACBP5 have been proven to confer salinity and pathogen resistance, respectively, in transgenic Arabidopsis and rice [93,94,127]. As various stress treatments induced the expression of *OsACBPs* [89], it would be interesting to identify their protein partners involved in these stress responses. In comparison to Arabidopsis, differential *GmACBP* expression in soybean upon salt and drought treatment suggests that soybean stress responses may differ from Arabidopsis [104], opening a new path to expand the study of leguminous GmACBPs in stress regulation. In view of the rapid growth in the global population, the discovery and knowledge of AtACBP-conferred stress resistance may be applicable in tackling food security issues related to crop protection in rice [89,93,94] and maize [103], and the production of engineered oil crops in sunflower [101,118] and oil palm [102]. The overexpression of ACBPs in developing seeds presents a molecular tool for the modification of nutritional and oil content in oil crops [85,130,131,180].

It is known that ACBPs play significant roles in plant development and stress responses [25,26,74,76,78,80,81,87,88,93,112,116,117,119,132], but the interplay between development and stress regulation is not well elucidated. For example, the overexpression of AtACBP1 not only plays a role in salt stress regulation [80], but also regulates seed dormancy and germination as well as seedling development through ABA signalling [78,80]. Nonetheless, their direct relationship is still not known, as complex signalling events of crosstalk between proteins, hormones and metabolites may be involved [181,182,183]. However, the use of lipidomics, proteomics, transcriptomics and other new technologies is expected to help unravel these comprehensive mechanisms to better understand their interactions.

## Figures and Tables

**Table 1:** Characterization of plant ACBPs.

Class	Protein Name	Signal Peptide	TM Domain	ACB Domain	Ankyrin Repeats	Kelch Motifs	Subcellular Locations	Size (kDa)
I	AtACBP6	−	−	+	−	−	Cytosol	10.4
	OsACBP1	−	−	+	−	−	Cytosol	10.2
	OsACBP2	−	−	+	−	−	Cytosol	10.3
	OsACBP3	−	−	+	−	−	Cytosol	17.7
	ZmACBP1	−	−	+	−	−	Cytosol	10.1
	HaACBP6	−	−	+	−	−	Cytosol, Nucleus	10.9
II	AtACBP1	−	+	+	+	−	ER, PM	37.5
	AtACBP2	−	+	+	+	−	ER, PM	38.5
	OsACBP4	+	+	+	+	−	ER	36
	ZmACBP3	−	−	+	+	−	ER	34.8
	EgACBP2	−	+	+	+	−	PM	ND
III	AtACBP3	+	+	+	−	−	Apoplast	39.3
	OsACBP5	+	+	+	−	−	ER	61.2
	ZmACBP6	−	−	+	−	−	Cytosol, PM	35.2
IV	AtACBP4	−	−	+	−	+	Cytosol	73.2
	AtACBP5	−	−	+	−	+	Cytosol	71
	OsACBP6	−	+	+	−	+	Peroxisomes	71.4
	ZmACBP7	−	−	+	−	+	Cytosol, PM	72.1

Abbreviations: ACB, acyl-CoA-binding; ACBP, acyl-CoA-binding protein; At, *Arabidopsis thaliana*; Eg, *Elaeis guineensis*; ER, endoplasmic reticulum; Ha, *Helianthus annuus*; kDa, kilodalton; ND, not determined; Os, *Oryza sativa*; PM, plasma membrane; TM, transmembrane; Zm, *Zea mays*; −, absent; +, present.

**Table 2:** Abiotic and biotic stress responses mediated by plant ACBPs and/or their interactors.

Proteins	Species	Acyl-CoA Binding	Phospholipid Binding	Protein Interactors	Stress Responses
AtACBP1	*A. thaliana*	16:0, 18:1, 18:2, 18:3, 20:4, 24:0, 25:0, 26:0 [26,108,133]	PC [78] PA [88]	PLDα1 [78]	Freezing [88]
				RAP2.12 [77,128,134]	Hypoxia [77,128]
				AREB1 [80]	Salinity, osmotic damage [80]
				−	Heavy metal [135]
				−	Pathogen [26]
AtACBP2	*A. thaliana*	16:0, 18:1, 18:2, 18:3, 20:4 [75,108,114]	PC [116] lysoPC [76]	AtEBP [73], RAP2.12 [77,128]	Hypoxia [73,77]
				LYSOPL2 [76,82], AtFP6 [75]	Heavy metal [75,76,82]
				−	Drought [90]
				−	Salinity [94]
				−	Oxidation [75]
AtACBP3	*A. thaliana*	12:0, 14:0, 16:0, 18:1, 18:2, 18:3, 20:4, 22:0, 24:0 [108,112,126,136]	PC [112] PE [112,117]	−	Drought [25]
				−	Hypoxia [136,137]
				−	Wounding [126]
				−	Pathogen [25,108,121]
AtACBP4	*A. thaliana*	14:0, 16:0, 18:0, 18:1, 18:2, 18:3 [113,115]	PC [115]	AtEBP [74]	Pathogen [25,74]
				−	Drought [25]
				−	Heavy metal [124]
AtACBP6	*A. thaliana*	14:0, 16:0, 18:0, 18:1, 18:2, 18:3, 20:4 [95,113,115]	PC [87]	−	Freezing [87,123]
				−	Drought [25]
				−	Wounding [125]
				−	Pathogen [25]
OsACBP4	*O. sativa*	16:0, 18:0, 18:1, 18:2, 18:3 [89,94]	PC, PA [91]	−	Salinity [89,94]
OsACBP5	*O. sativa*	16:0, 18:3 [89,93]	PC, PA [91]	−	Pathogen [89,93,127]
				−	Wounding [89]
OsACBP6	*O. sativa*	18:1, 18:2 [89]	PC, PA [91]	−	Wounding [89]
ZmACBP1	*Z. mays*	−	−	−	Salinity, drought [103]
ZmACBP3	*Z. mays*	−	−	−	Salinity, drought [103]
ChACBP1	*Chlorella* sp.	−	PC [92]	−	Freezing, salinity, oxidation, heavy metal [92]
VvACBP	*V. vinifera*	−	−	−	Freezing, heat, ER, pathogen [100]

Abbreviations: AREB1, ABSCISIC ACID-RESPONSIVE ELEMENT BINDING PROTEIN1; AtEBP, Arabidopsis ETHYLENE-RESPONSIVE BINDING PROTEIN; AtFP6, Arabidopsis FARNESYLATED PROTEIN6; ER, endoplasmic reticulum; lysoPC, lysophosphatidylcholine; LYSOPL2, LYSOPHOSPHOLIPASE2; PA, phosphatidic acid; PC, phosphatidylcholine; PE, phosphatidylethanolamine; PLDα1, PHOSPHOLIPASE Dα1; RAP2.12, RELATED TO APETALA2.12.

## Data Availability

Not applicable.
